# Mutation analysis of exon1 of *bone morphogenetic protein-15* gene in Iranian patients with polycystic ovarian syndrome

**Published:** 2016-08

**Authors:** Anahita Mehdizadeh, Mohammad Hasan Sheikhha, Seyed Mehdi Kalantar, Bibi Shahnaz Aali, Azam Ghanei

**Affiliations:** 1 *Biotechnology Research Center, International Campus, Shahid Sadoughi University of Medical Sciences, Yazd, Iran.*; 2 *Department of Medical Genetics, Research and Clinical Center for Infertility, Shahid Sadoughi University of Medical Sciences, Yazd, Iran.*; 3 *Physiology Research Center, Department of Obstetrics and Gynecology, Afzalipour Hospital, Kerman University of Medical Sciences, Kerman, Iran.*; 4 *Department of Internal Medicine, Shahid Sadoughi University of Medical Sciences, Yazd, Iran.*

**Keywords:** *BMP-15*, *PCOS*, *Mutation*, *Sequencing*

## Abstract

**Background:**

With the prevalence of 6-10%, polycystic ovarian syndrome (PCOS) is considered the most common endocrinological disorder affecting women in their reproductive age. It has been suggested that genetic factors participate in the development of PCOS. Follicular development has been considered as one of the impaired processes in PCOS. Bone morphogenetic protein-15 (*BMP-15*) gene is a candidate gene in follicular development and its variants may play role in pathogenesis of PCOS.

**Objective:**

To investigate whether *BMP-15* gene mutations are present in Iranian women with PCOS.

**Materials and Methods:**

In this cross-sectional study 5 ml venous blood samples was taken from 70 PCOS women referring to Afzalipour Hospital, Kerman University of Medical Sciences, Kerman, Iran, between January to December 2014. Genomic DNA was extracted from the blood sample by salting out method. Then a set of PCR reactions for exon1 of *BMP-15* gene was performed using specific primers followed by genotyping with direct sequencing.

**Results:**

Two different polymorphisms were found in the gene under study. In total 20 patients (28.6%) were heterozygote (C/G), and 2 patients (2.86%) were homozygous (G/G) for c.-9C>G in 5´UTR promoter region of *BMP-15* gene (rs3810682). In addition, in the coding region of exon1, three patients (4.3%) were heterozygote (G/A) for c.A308G (rs41308602). Two PCOS patients (2.86%) appeared to have both c.-9C>G (C/G) and c.A308G (G/A) variants simultaneously.

**Conclusion:**

Our research detected two polymorphisms of *BMP-15* gene among PCOS patients, indicating that even though it cannot be concluded that variants of *BMP-15* gene are the principal cause of polycystic ovarian syndrome; they could be involved in pathogenic process in development of PCOS.

## Introduction

Polycystic ovarian syndrome (PCOS) is globally known as the most common endocrine disorder affecting 5-10% of reproductive women. PCOS is diagnosed by hyperandrogenism, infertility, menstrual irregularities and polycystic ovaries (1). Additionally, individuals with PCOS might be at the risk of metabolic disorders, obesity and even endometrial cancer which has severe life threatening effects (2). 

Despite the uncertainty in PCOS etiology, studies which aimed to find molecular basis and genetic risk factors of PCOS resulted in discovering a number of genes which are likely to be impaired in PCOS. Recent investigations focusing on anovulatory condition in PCOS women, reported possible role of oocyte secreted factors, in particular, bone morphogenetic protein 15 (BMP-15), a member of transforming growth factor β (TGFβ) superfamily (3). 

This protein factor have significant roles in development of follicles, oocyte maturation and embryo development (4, 5). In situ hybridization and immunohistochemical analysis have shown the expression of BMP-15 in human oocyte throughout follicular development. Previous investigations have reported the possibility of* BMP-15* as a candidate gene in PCOS due to its local modulator of ovarian function. *BMP-15* is located on Xp11.2 and is consisted of two exons (6). 

The role of *BMP-15* gene has been tested in animal models. In *BMP-15* null mice, primary defects in ovulation and fertility with subsequent reduction in female fertility were observed. Moreover, in sheep, BMP-15 plays role as a breeding marker that affects ovulation rate (4, 5, 7).

Since there has been no evidence of association of BMP-15 and PCOS population in Iranian women, we designed this study to investigate whether any mutation is present in the Iranian PCOS women in Kerman province.

## Materials and methods

In total 70 PCOS women aged 18-40 were recruited in this study. The PCOS was confirmed based on Rotterdam criteria which necessitate presence of at least two of PCOS characteristics for selection. Other causes of hyperandrogenism such as Cushing’s syndrome were excluded. 

The standard salting out method was used to extract genomic DNA from peripheral blood leukocytes. The blood sample was chosen for this study instead of oocyte because the mutation in the genome was the subject of the study and not the gene expression. After DNA extraction, amplification of exon1 of *BMP-15* gene in all PCOS individuals was performed by polymerase chain reaction (PCR) using a set of primer: forward 5’-CATGCTGCCTTG TCCCACCTTC-3’ and reverse 5’-GTAGGC TTGGCGCTGGCTTCTC-3’ which amplifies a 742 bp segment of DNA.

**Figure 1:**
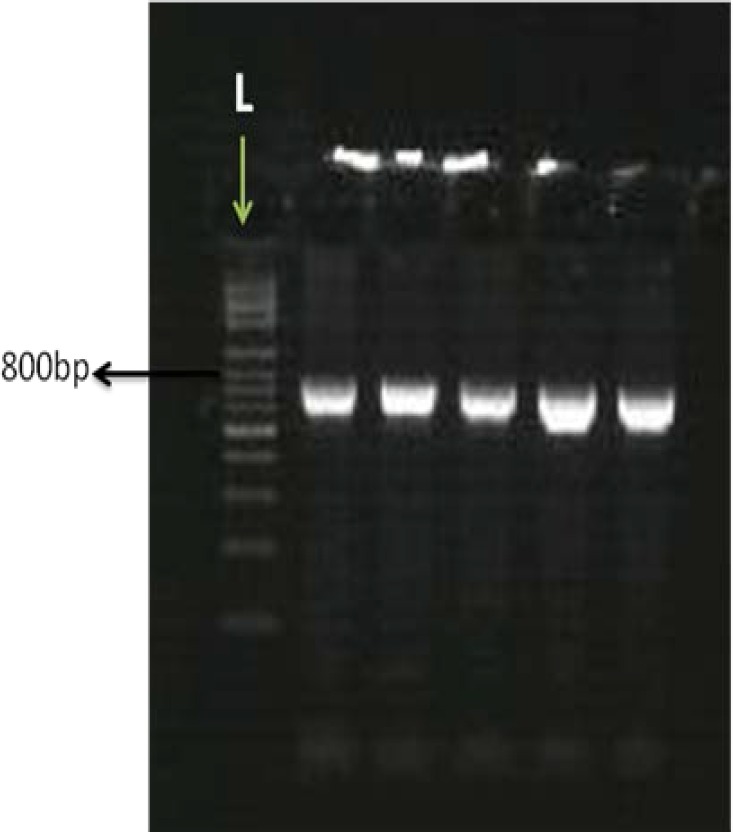
The amplified bands are 742bp and consisted of exon1 of *BMP15* on 1.5% agarose gel L: ladder

**Figure 2:**
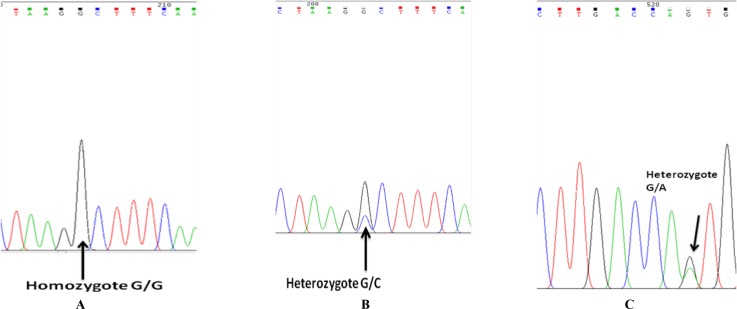
SNP rs3810682 (c.-9C>G) in promoter region of *BMP-15* gene, A; the homozygote form (G/G), B; the heterozygote form (G/C) and rs41308602 (A308G),  C; the heterozygote form (G/A) in SNP site

PCR reactions were consisted of 35 cycles of denaturation (95^o^C for 30 sec), annealing (66^o^C for 30 sec) and extension (72^o^C for 30 sec). The initial step for denaturation was 5 minutes of 95^o^C and the final segment of the PCR (final extension) was 5 min of 72^o^C. The resulted PCR products were tested on agarose gel (Figure 1) for ideal concentration and sequenced by an automated sequencer (ABI 3730; Applied Biosystems) in order to perform mutation analysis (Figure 2).

The study was approved by committee of ethics in Medical Faculty, Shahid Sadoughi University of Medical Sciences, Yazd, Iran. Written informed consent was obtained from all participants.

## Results

In the first exon of BMP-15, two variants were found which were reported in earlier studies: c.-9C>G in 5’UTR, specifically in promoter region (rs3810682) and c.A308G in coding region of exon1 (rs41308602). In the latter there is a substitution of serine instead of asparagine.

The -9C>G in the promoter region was detected in 22 patients (31.4%) including 20 cases (28.6%) of heterozygote (C/G) and 2 cases (2.86%) of homozygote form of this variant (G/G). For calculating the allele frequency, two cases of homozygote (G/G) each had two allele and 20 heterozygote cases each had one allele, resulting in 24 allele all together (Table II). 

Furthermore, three cases (4.3%) of heterozygote form (G/A) for c.A308G were revealed and because there was no homozygote G/G genotype therefore the allele count for G allele would be three as well (Table III). Among those cases, two patients (2.86%) had both c.-9C>G and c.A308G variants.

**Table I:** Summary of the mutations found in *BMP15* gene in this study

**SNP**	**a/a**	**Genotype**	**Position**	**Number**
-9 C>G	C/G	Heterozygote	5'UTR	18
-9 C>G	G/G	Homozygote	5'UTR	2
A308G	G/A	Heterozygote	Exon1	1
-9 C>G	C/G	Heterozygote	5'UTR	2
A308G	G/A	Heterozygote	Exon1	

SNP: Single nucleotide polymorphism

a/a: Aminoacids

**Table II:** Frequency distribution of different genotypes and alleles of rs3810682 (c.-9C>G)

**Alleles**	**C**	**G**	**C/C**	**C/G**	**G/G**
Number (%)	116 (82.9)	24 (17.1)	48 (68.6)	20 (28.6)	2 (2.9)

**Table III:** Frequency distribution of different genotypes and alleles of rs41308602 (A308G

**Alleles**	**A**	**G**	**A/A**	**A/G**	**G/G**
Number (%)	137 (98)	3 (2)	67 (95.7)	3 (4.3)	0 (0)

## Discussion

BMP-15 is initially a preprotein which later, after cleavage, forms a homodimer or heterodimer with growth differentiation factor 9 (GDF9), another member of TGFβ superfamily which is secreted from oocyte (8). Coexpression of these two factors in human oocyte plays a crucial role in molecular connection between oocyte and the other peripheral somatic cells which consequently encourage proliferation of granulosa cells and cumulus expansion by a paracrine effect (9, 10). 

Regulation of folliculogenesis is one of the crucial roles of oocyte in mammalian ovary. Oocytes particularly affect granulosa cells and stimulate their proliferation, modulate gene expression in granulosa cells, regulate formation of follicles prenatally and affect steroidogenesis. BMP-15 which is expressed in human oocyte, might be essential for development of preantral follicle. Even though earlier investigations regarding mutational analysis of *BMP-15* gene were associated with ovarian failure, reports on *BMP-15* gene in PCOS are also available (11-15). 

In a Japanese study, 38 PCOS women were screened, though the study did not show any missense mutation (16). In another report based on association analysis of *BMP-15* gene in a population of Spanish women, results failed to show any association between PCOS and *BMP-15* gene mutations (17). The Chinese analysis was the first one that provided evidence of association of *BMP-15* gene and PCOS by discovering 7 variants in *BMP-15* gene which 5 of those were novel missense mutations (18).

Analyzing the naturally occurred mutations in sheep revealed that mutations make aberrations in natural folding of BMP-15 which consequently leads to formation of unstable heterodimers and homodimers that are eventually subjected to degradation. Incorrect formation of heterodimers and homodimers result in reduced level of normal BMP-15 dimer levels and alteration in BMP-15 expression during development of follicles (11). *BMP-15* mutations may be important in cessation of follicle development and hence results in accumulation of small antral follicles and development of polycystic ovary phenotype. 

In the present study we found two variants, -9 C>G and A308G in 23 PCOS patients. In two patients both of these polymorphisms happened simultaneously. These polymorphisms were reported by other researchers either in PCOS patients or in the other diseases (19-23). In our results A308G polymorphisms occurred in three patients out of 70 PCOS participants, while in Liu *et al *study, only one case of this polymorphism in 216 PCOS patients were reported (18). The difference in the prevalence of mutations might be because of ethnic/genetic differences between Chinese and Persian population.

## Conclusion

In conclusion this study is the first one to conduct on Iranian population of PCOS patients to verify the polymorphisms in *BMP-15* gene and the mentioned polymorphisms were found in the population under study. Finding these polymorphisms of *BMP-15* gene among PCOS patients, indicates that even though it cannot be concluded that variants of *BMP-15* gene are the principal cause of polycystic ovarian syndrome; they could be involved in pathogenic process of development of PCOS. 

Further investigations are needed to do biochemichal analysis of hormones on patients with mutations. Moreover, further studies needed to be performed on normal cases and on different populations to establish whether *BMP-15* can be served as a genetic marker for PCOS.

## note

***This article extracted from M.Sc. thesis. (Anahita Mehdizadeh)***
